# Insights on the Adsorption of Per- and Polyfluoroalkyl Substances onto Laboratory Syringe Membrane Filters: Experimental, Materials, and Mechanism Evaluations

**DOI:** 10.1021/acsestwater.5c00438

**Published:** 2026-03-07

**Authors:** Elliot Reid, Qingquan Ma, Thomas Igou, Ching-Hua Huang, Yongsheng Chen

**Affiliations:** † School of Civil and Environmental Engineering, 1372 Georgia Institute of Technology, Atlanta, Georgia 30332, United States; ‡ Water Tectonics Inc, 6300 Merrill Creek Pkwy C-100, Everett, Washington 98203, United States

**Keywords:** per- and polyfluoroalkyl substances (PFAS), filtration, adsorption, membranes, materials characterization, mechanisms of adsorption

## Abstract

When samples containing perfluoroalkyl and polyfluoroalkyl substances (PFAS) are filtered prior to analysis, inadvertent adsorption onto membrane materials can result in concentration underestimations. Herein, we systematically examined the adsorption of six PFAS (PFOA, PFOS, PFNA, PFHxS, PFBS, GenX) onto 11 membrane syringe filters, differing in manufacturer, polymer material, diameter, and/or pore size under various experimental conditions. We perform a comprehensive characterization of the membrane materials to determine differences in morphology, zeta potential, porosity, surface area, and roughness. Evaluation of postfiltration PFAS recovery demonstrated significant impacts of filter material and surface area (related to pore size and diameter), initial PFAS concentration, water pH, and the co-occurrence of cations and anions. Longer-chain PFAS more readily adsorb than shorter-chain PFAS for all filter materials under all experimental conditions. Machine learning predictions of Abraham’s solute descriptors were used to qualitatively assess the predominant forces governing PFAS adsorption onto different materials. While differences in hydrogen-bonding ability exert influence, hydrophobicity and electrostatic interactions are the main drivers of adsorption, along with significant impacts of the water matrix. We recommend polypropylene, mixed cellulose ester, or glass fiber filters with larger pore sizes and smaller diameters to filter PFAS-containing samples, and we discourage the use of nylon-based filters. These findings offer important insights for optimizing PFAS sample preparation and improving membrane design for effective PFAS removal.

## Graphical abstract

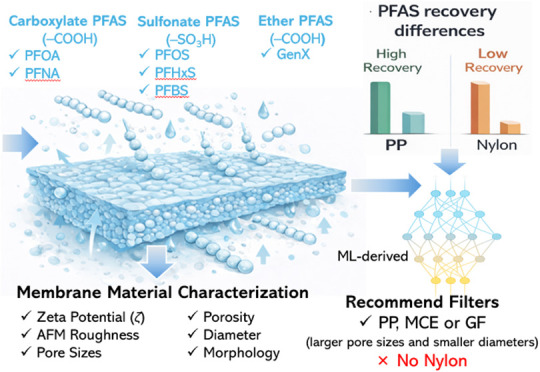

## 1 Introduction

Per- and polyfluoroalkyl substances (PFAS) are a synthetic chemical class comprised of more than 15,000 different compounds that are unified by the notoriously difficult-to-break carbon–fluorine bond (bond dissociation energy, 105.4 kcal/mol).^1^ PFAS are omnipresent in the environment, have been detected globally in water, soil, and air,^2–5^ and are associated with increased morbidity risks for humans and detrimental effects to various environmental biota.^6–8^ In 2023, the U.S. Environmental Protection Agency (EPA) proposed the National Primary Drinking Water Regulation (NPDWR) to reduce the long-term exposure to PFAS in drinking water.^9^ Specifically, the NPDWR targets six perfluorocarboxylic acids (PFCAs) and perfluorosulfonic acids (PFSAs): namely, perfluorooctanoic acid (PFOA), perfluorononanoic acid (PFNA), perfluorooctanesulfonic acid (PFOS), perfluorobutanesulfonic acid (PFBS), perfluorohexanesulfonic acid (PFHxS), and hexafluoropropylene oxide dimer acid (HFPO–DA, more commonly known as GenX)—with the goal of establishing more stringent maximum contaminant-level goals (MCLGs). In 2024, EPA finalized the regulation by setting the maximum contaminant levels (MCLs) at 4 ng/L each for PFOS and PFOA, 10 ng/L each for PFNA, PFHxS, and GenX, and a Hazard Index of 1.0 for combined PFNA, PFBS, PFHxS, and GenX.^10^

Due to the increasingly stringent concentration levels set on PFAS, it is imperative that they are measured as accurately as possible to properly determine and report risk. Unfortunately, due to their amphiphilic physicochemical nature, aqueous PFAS contact with various laboratory materials during sampling and analysis can result in unintentional adsorption, leading to concentration-level underestimations. A key vector for fugitive PFAS adsorption is the routine use of disposable laboratory membrane syringe filters, which are commonly employed to remove solid particles from liquid samples to protect sensitive analytical equipment (e.g., mass spectrometers) from increased maintenance, repair, and/or replacement of parts caused by particle introduction. There are a myriad of membrane syringe filters available for purchase from many different vendors, differing in membrane materials, manufacturer, pore size, and diameter, which can produce differences in experimental results (i.e., if different filters are used in different experiments of the same study, the results can be wildly different). EPA methods 1633, 533, and 537.1 pertain to the measurements of different PFAS compounds in various environmental matrices such as wastewater, surface water, drinking water, soil, *etc*. Method 1633 requires that the sample extract is filtered after undergoing solid-phase extraction (SPE) concentration (not on the raw sample), while methods 533 and 537.1 specifically prohibit filtration either before or after SPE. Additionally, many laboratory studies do not mimic environmental sampling; for example, adsorption studies utilize a filtration step to separate solid adsorbent from the aqueous stream.^11^ There is also literature evidencing the converse of PFAS concentration underestimation, showing that measured PFAS can be artificially high due to laboratory contamination, analytical interference, and sample preparation.^12,13^

A handful of studies have investigated the inadvertent PFAS adsorption onto various laboratory materials. Lath et al. examined the sorption losses of PFOA onto centrifuge tubes constructed from PP, polystyrene (PS), glass, and polycarbonate (PC), finding wide variation of sorption losses under different conditions, with proportional losses of PFOA decreasing with increasing PFOA concentrations.^14^ Sörengård et al. conducted a systematic study examining the loss of 21 different PFAS to membrane syringe filters in deionized (DI) water, methanol, and water containing organic carbon, finding the highest and lowest losses of PFAS to filters in DI water and methanol, respectively, and finding greater adsorption of longer-chain PFAS to filters.^15^ Chandramouli et al. investigated the adsorption of 26 different legacy and less-conventional PFAS (*e*.*g*., fluorotelomers, perfluoroalkyl phosphate diesters) in several aqueous matrices onto glass fiber (GF), nylon, polyether sulfone (PES), and PTFE filters, finding high adsorption losses of most PFAS to both nylon and PTFE.^16^ Interestingly, hydrophobic-PTFE filters have been reported to offer the lowest mass losses during filtration for a large number of pharmaceuticals and personal care products (PCPPs).^17^ Zheng et al. assessed the sorption of PFOA, PFOS, and PFBA onto six syringe filters of differing materials in both DI water and wastewater effluent; the authors found PFBA to adsorb the least readily of the three PFAS, mixed cellulose ester (MCE) to be the best filter candidate, and PTFE to be the worst filter material (likely due to fluorophilic interactions).^18^ Morishita et al. examined sorption of nine PFAS of different chain length onto 11 different filter materials, finding high recoveries of all PFAS for all materials in methanol, increasing PFAS recoveries with increasing filtered solution volumes, and poor PFAS recoveries for nylon membranes.^19^ The choice of bottle material used for containing liquids is also important. Zenobio et al. described the general trend of adsorption from a solution containing PFAS to follow, in order of lowest adsorptive losses to highest, polypropylene (PP), high-density polyethylene (HDPE), polyethylene terephthalate (PET), glass, and PS, agreeing with EPA Method 537.1′s container choice for PFAS analysis.^20^ Unfortunately, these phenomena add complexity to the already complex PFAS problem and warrant further investigation.

While there are some literature data available regarding PFAS adsorption onto laboratory materials such as syringe filters, few studies have truly attempted to explain the physical and chemical mechanisms driving the differences in PFAS recovery for different materials. Machine learning (ML), simulation methods, and other computational efforts have been useful in providing deeper knowledge regarding the behavior of PFAS at material and medium interfaces. For instance, Jeong et al. utilized a combination of Shapley additive explanation techniques and molecular dynamics (MD) simulations to emphasize the dominance of electrostatic interaction during PFAS transport across polyamide membranes and also the importance of the PFAS functional group in regulating transport.^21^ Sleep et al. conducted Monte Carlo molecular simulations of PFOS and PFBS interacting with surfaces of various surface charge in the presence of H^+^, OH^–^, and Ca^2+^, finding that positively charged surfaces encourage greater adsorption than that of negatively charged ones even in the presence of ions; they also found that cationic bridging of PFAS with negatively charged graphite surface facilitated higher adsorption.^22^

Another tool to hypothesize the driving mechanisms behind PFAS adsorption onto different surfaces concerns the examination of polyparameter linear free energy relationships (LFER). LFERs can be used to predict equilibrium adsorption of a compound between two distinct phases (*e*.*g*., octanol/water) and provide mechanistic insights of molecular interactions between absorbents and absorbates by Abraham’s solute descriptors (ASD) (*e.g*., excess molar refraction, polarizability, hydrogen bond acidity and basicity, and McGowan characteristic volume).^23–26^ There are a few LFER models that describe adsorption behavior at a water/polymeric surface interface (*e.g*., low-density polyethylene),^27^ and to the best of our knowledge, none that concern the materials we test in this work. Examination of the ASD can be useful in probing and developing hypotheses regarding adsorption mechanisms. Researchers at the Massachusetts Institute of Technology (MIT) and Northeastern University developed a free, open-source software package, Reaction Mechanism Generator (RMG), that is capable of using an ML method to predict ASD.^28–30^ Previous works have used RMG for various purposes, including kinetic modeling of chemical reactions.^31–33^

Herein, we add to the small handful of studies examining PFAS adsorption onto commonly utilized laboratory materials by performing comprehensive materials characterization, investigating new experimental conditions, and describing the mechanisms that result in different PFAS recoveries during filtration. Specifically, we report on the adsorption of six PFAS—namely, PFOA, PFOS, PFBS, PFHxS, PFNA, and GenX—onto a variety of different common membrane syringe filter materials. We elucidate how key parameters (*e.g*., diameter, pore size) of many comparable filters (*i.e*., created by the same manufacturer) result in differences in adsorption behavior across a variety of experimental test conditions (*e.g*., pH) and link these observations to material properties. We then utilize RMG’s ML-predicted ASD of both PFAS and polymeric monomers/dimers to describe the mechanisms of PFAS adsorption onto individual filter materials, highlighting key intermolecular forces that govern the adsorption process. This work adds new information to the complexity of PFAS adsorption onto different surfaces, better informs experimental design and reporting, and could be a valuable tool in the design of membranes for PFAS-specific separations.

## 2 Materials and Methods

### 2.1 Chemical Reagents and Materials

All PFAS were purchased from Sigma-Aldrich (Burlington, MA) with the exception of GenX, which was purchased from Toronto Research Chemicals (Toronto, Canada); PFAS structure and physicochemical properties are detailed in Supporting Information (SI) Table S1. The following chemicals were also purchased from Sigma-Aldrich for ionic addition/pH change experiments: reagent-grade FeCl_3_*6H_2_O, CaCl_2_*2H_2_O, Na_2_SO_4_, Na_2_HPO_4_/NaH_2_PO_4_, MgCl_2_, NaCl, HCl, and NaOH. A variety of filter materials were used for this study, and their various physical properties are detailed in Table 1; the chemical structures of these materials can be found in SI Figure S1. Materials were selected to account for both similarities and differences in manufacturer, polymeric material, chemical functional groups, diameter, and pore size. No fluorinated materials were used to avoid fluorophilic interactions. For all filtration experiments, the filters were connected to a 3 mL polypropylene syringe (Lite Touch) via Luer lock fittings. Reagent-grade DI water with a resistivity of 18.2 MΩcm^–1^ and total organic carbon (TOC) concentrations ranging from 5 to 25 parts-per-billion (ppb) was produced by a water purification system (Milli-Q Benchtop Lab Water Purification System, Millipore Sigma, Burlington, MA).

**Table 1:** Filter Material Details

filter code	filter material	manufacturer	pore size (μm)	diameter (mm)
A	Polypropylene (PP)	Foxx Life Sciences	0.22	13
B	Polyether sulfone (PES)	Biomed Scientific	0.22	13
C	Polyether sulfone (PES)	Biomed Scientific	0.22	25
D	Polyether sulfone (PES)	Biomed Scientific	0.45	13
E	Polyether sulfone (PES)	Biomed Scientific	0.45	25
F	Cellulose Acetate (CA)	Sterlitech	0.45	13
G	Nylon	VWR	0.22	25
H	Nylon	VWR	0.22	13
I	Nylon	Sterlitech	0.45	25
J	Mixed Cellulose Ester (MCE)	Fischer-Scientific	0.45	33
K	Mixed Cellulose Ester (MCE)	Omicron	0.45	25
L	Glass Fiber (GF)	Tisch Scientific	0.45	25

### 2.2 Material Characterization

Membranes were carefully extracted from their plastic housing via mechanical methods for property characterization. Scanning electron microscopy (SEM) was performed using a Hitachi SU8230 (Tokyo, Japan) operating at 5.0 kV at an 8 mm working distance to visualize filter morphology. Each sample was coated with a thin layer of gold and was placed on a conductive tape prior to imaging. Surface roughness was analyzed using an atomic force microscope (AFM, Bruker Dimension Icon, MA, USA) in tapping mode with a scanning range of 20 μm × 20 μm on a membrane coupon under ambient conditions. A sampling resolution of at least 256 points per line and a speed of 0.1–0.5 Hz were used, and AFM analysis software was used to calculate surface roughness. The membrane surface zeta potential was determined using a Zetasizer electrokinetic analyzer (Malvern Instruments, U.K.) with 1 mM KCl as the electrolyte solution. The pH was adjusted by using either NaOH (0.1 M) or HCl (0.1 M) solutions. Two PES membrane filters of the same manufacturer and diameter, but differing pore sizes, were shipped to the Particle Testing Authority (Norcross, GA, U.S.) for mercury intrusion porosimetry measurements using an AutoPore IV 9500 2.03.00.

To calculate porosity, membrane samples were immersed in DI water for 4 h; afterward, the membranes were removed from water and were weighed after mopping superficial water with filter paper. The wet membranes were then placed in an air-circulating oven at 60 °C overnight and then further dried in a vacuum oven at 80 °C for 24 h before measuring the dry weight. From the two weights (wet sample weight and dry sample weight), the porosity of membrane was calculated using eq 1, where *P* is the porosity of the membrane, *Q*_0_ is the wet sample weight (g), *Q*_1_ is the dry sample weight (g), *A* is the area of the membrane (cm^2^), and *h* is the thickness of the membrane (mm, determined via SEM cross section information).

1 $$P\;\left(\%\right)=\frac{Q_{0}-Q_{1}}{\mathrm{Ah}}\times 1000$$

### 2.3 Experimental Methods

A glass beaker containing approximately equal concentrations of each of the six PFAS in different water matrices was prepared and mixed via a stir bar rotating at 200 rpm. Controls with no sample filtration were taken to measure unfiltered PFAS concentrations and gauge PFAS recovery. For filtration experiments, 1 mL of the PFAS-containing mixture was pipetted into a syringe with a filter attached. Filtration of the solution through the filter and into a glass autosampler vial was standardized to the best of our ability by time (on average, 2 s) and intensity. Each sample was taken in duplicate by using a new syringe and filter for each filtration event. To examine the effect of pH, DI water containing known concentrations of added PFAS was adjusted to either acidic or basic levels using HCl or NaOH, respectively. For experiments containing ionic addition, known amounts of an ion of interest were added by using concentrated stocks. Collected samples were stored at 5 °C until analysis. The filters were evaluated in terms of percent recovery of PFAS, eq 2, where a greater recovery value means that a lesser amount of the PFAS adsorbs to the filter material.

2 $$\mathrm{recovery}\;\left(\%\right)=\frac{C_{F}}{C_{0}}\times 100$$

where *C*_0_ and *C*_F_ are the concentrations of an individual PFAS molecule in the unfiltered control sample and in the filtered sample, respectively. It was assumed that 100% of the difference in PFAS concentrations was caused by adsorption of the material to filter materials.

### 2.4 Analytical Quantification

PFAS concentrations were analyzed using high-performance liquid chromatography tandem mass spectrometry (HPLC-MS/MS) (Agilent Technologies, 6410 Triple Quad LC/MS) equipped with an Agilent Poroshell EC-18 column (4.0 μm particle size, 2.1 mm × 150 mm). The instrument was operated in an electrospray ionization (ESI) negative mode. The dual solvent mobile phase consisted of varying ratios of 5 mM ammonium acetate in LC/MS-grade water (phase A) and 80/20 v/v LC/MS-grade methanol and acetonitrile (phase B) at a constant flow rate of 0.25 mL/min. The injection volume was 20 μL, and the total run time was 24.5 min. Optimal separation of PFAS compounds was achieved with a gradient of 90% A (0–2) min, 30% A (2–4) min, 2% A (4–18 min), and 100% A (18–24.5 min), with the balance provided by B. Calibration standards were created for each experimental condition, ranging from 5 ppb to 500 ppb of PFAS; in cases where substantial matrix effects were observed compared to the DI control, PFAS standards were created in that test matrix.

### 2.5 Collection of Abraham Solute Descriptors

ASD for the PFAS and monomers/dimers of the membrane materials were predicted using the open-source Reaction Mechanism Generator (RMG) software.^28^ ASD (*i.e*., *E*, *S*, *A*, *B*, *L*, *and V*) were predicted by input of a compound’s simplified molecular input line entry system (SMILES) into the RMG solute parameter search or prediction solvation tool using the Solute ML method. The Solute ML method predicts a compound’s ASD from a database containing 8,366 solute molecules with known ASD and 195 solvents with fitted Abraham solvent parameters (*c*, *v*, *e*, *s*, *a*, *b*, *l*). ASD are used to predict a compound’s adsorption behavior in a particular interface (*e.g*., air, solvent, solid), by relating physicochemical properties to established LFERs (eq 3) (*e.g*., the octanol/water LFER).

3 $$\log K_{i12}=c+v_{12}V_{i}+e_{12}E_{i}+s_{12}S_{i}+a_{12}A_{i}+b_{12}B_{i}$$

where log *K*_i12_ is the partitioning coefficient for compound *i* between phases 1 and 2 at equilibrium conditions, *c* is the regression constant, *e*_12_, *s*_12_, *a*_12_, *b*_12_, and *v*_12_ are fitting coefficients unique to phases 1 and 2, *E*_*i*_ is the excess molar refraction ((cm^3^ × mol^–1^)/10) that represent the contribution of van der Waals forces (which is sometimes replaced with *L*_*i*_, the log of gas/*n*-hexadecane partitioning coefficient, *S*_*i*_ is the polarizability parameter (*i.e*., dipole-type interactions), *A*_*i*_ and *B*_*i*_ are the hydrogen bond donating and accepting abilities, respectively, and *V*_*i*_ is McGowan’s volume ((cm^3^ × mol^–1^)/100)).

## 3 Results and Discussion

### 3.1 Materials’ Characterization

Comprehensive characterization of the filter membrane materials demonstrated notable differences, as compiled in Table 2. Figure 1 shows representative AFM scans and SEM images for filter samples (A) PP 0.22 μm, 13 mm, (E) PES, 0.45 μm, 25 mm, (F) CA, 0.45 μm, 13 mm, and (L) GF, 0.45 μm, 25 mm; images and scans for other filters are included in SI Figures S2 and S3. Two predominant material morphologies were observed from SEM, namely, (1) porous disc, seen by (E) and (F), and (2) strand-like, seen by (A) and (L). AFM scans show a wide range of roughness values (*i.e*., 71 nm (D) −894 nm (L), RMS roughness). Unsurprisingly, the irregular interconnected fibers of the strand-like filters have relatively greater surface roughness compared to the more uniform porous disc structures.

**Table 2:** Characterization Data of the Filter Materials

code	material	porosity (%)	zeta potential (mV)	root mean square roughness (Rq, nm)	average roughness (Ra, nm)	morphology (SEM)
A	Polypropylene (PP)	28.8	–43 ± 2.5^a^	467	355	Strand-like
B	Polyether sulfone (PES)	31.25	–53 ± 3.1^a^	107	77.5	Porous disc
C	Polyether sulfone (PES)	34.25	–50 ± 2.7^a^	137	94.4	Porous disc
D	Polyether sulfone (PES)	35.64	–48 ± 3.4^a^	71.1	50.7	Porous disc
E	Polyether sulfone (PES)	42.57	–49 ± 2.8^a^	245	161	Porous disc
F	Cellulose Acetate (CA)	44.78	–42 ± 3.2^a^	62.8	45.9	Porous disc
G	Nylon	52.75	–21 ± 2.1^a^	201	159	Porous disc
			–16 ± 3.2^b^			
			–35 ± 2.7^c^			
H	Nylon	54.56	–24 ± 1.9^a^	474	367	Porous disc
			–14 ± 1.2^b^			
			–36 ± 3.4^c^			
I	Nylon	33.21	–23 ± 1.2^a^	463	353	Porous disc
			–13 ± 2.5^b^			
			–43 ± 4.5^c^			
J	Mixed Cellulose Ester (MCE)	42.52	–42 ± 2.4^a^	691	553	Porous disc
K	Mixed Cellulose Ester (MCE)	46.58	–45 ± 3.1^a^	230	182	Porous disc
L	Glass Fiber (GF)	31.47	–38 ± 2.6^a^	894	670	Strand like

a pH 6.5.

b pH 3.9.

c pH 9.3.

**Figure 1:**
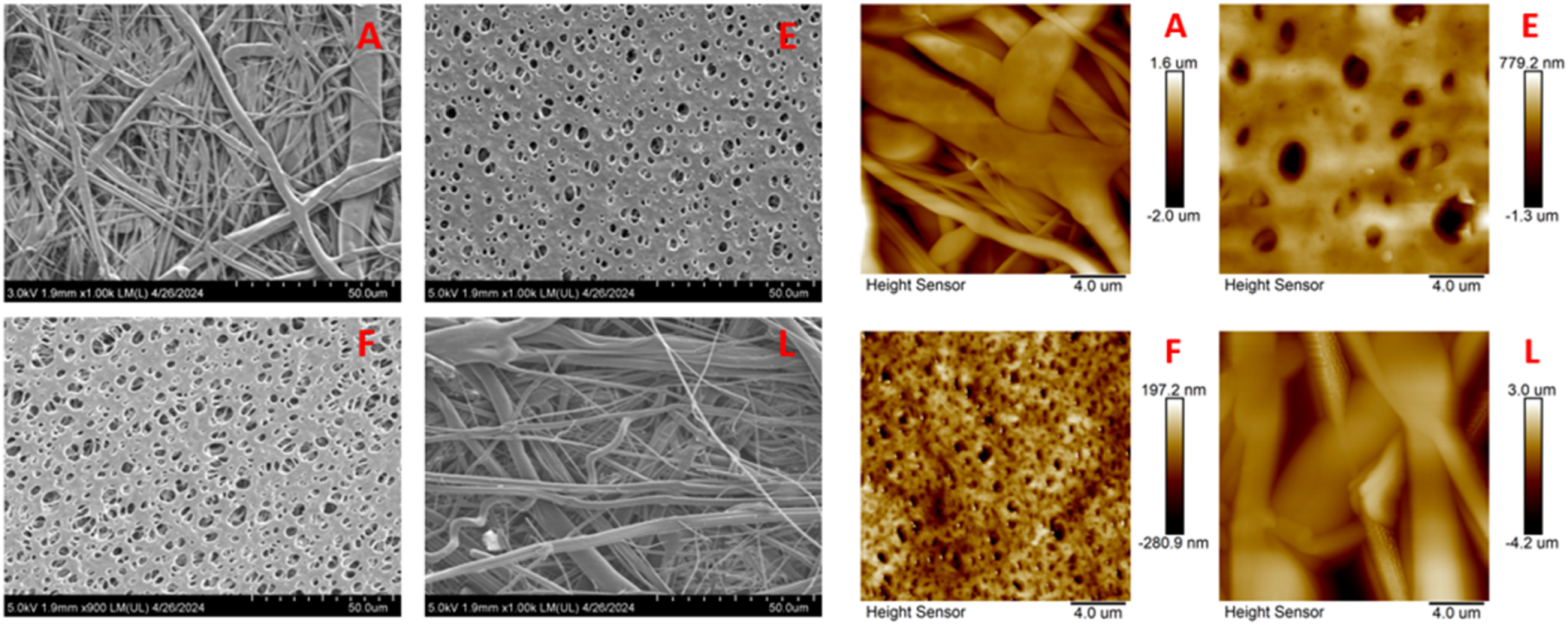
Representative SEM images (50 μm resolution) and AFM topographical scans of (A) PP 0.22 μm, 13 mm; (E) PES, 0.45 μm, 25 mm; (F) CA, 0.45 μm, 13 mm; (L) GF 0.45 μm, 25 mm.

Zeta potential measurements at pH 6.5 were taken for all filter types, revealing that all of the materials have inherently negatively charged surfaces at near-neutral pH, which would suggest anionic PFAS repulsion due to unfavorable electrostatic interactions. These zeta potential values are only slightly different for the majority of the materials with the exception of the nylon samples, which are significantly more positively charged than the other materials at near-neutral pH. Additional zeta potential measurements were made for these nylon samples at both pH 4.0 and 9.0 to support experimental observations, which we discuss later in Section 3.3; it is clear that the nitrogenous character of nylon significantly impacts the zeta potential at different pH ranges due to the degree of protonation of amine groups present at the end of the polymer branches (p*K*_a_ ∼ 9–10).

Porosity measurements can be compared for the four PES filters and two of the three nylon filters since they have the same filter diameter. As clearly seen in the differences between filters B and D, filters C and E, and filters G and I, the porosity expectedly increases as the pore size of the material increases. Differences in porosity among the filter materials in general could arise from manufacturer fabrication techniques although comparable filters with larger pore sizes likely have less overall available surface area to facilitate adsorption. Mercury intrusion porosimetry (MIP) was performed on PES membrane filters (B) and (D), which were created by the same manufacturer and have the same diameter but differing pore sizes. Expectedly, results indicate that the 0.45 μm filter has less overall surface area compared to the 0.22 μm filter (total intrusion volume 2.3761 vs 2.6839 (mL/g), total pore area 49.09 vs 61.82 (m^2^/g), and average pore diameter 0.1936 vs 0.1737 (μm) for PES 0.45 μm and PES 0.22 μm, respectively) (SI Table S2).

### 3.2 Effect of Surface Area and PFAS Concentration on Recovery

Figure 2 illustrates the percent recovery for each individual PFAS from DI water solution by 11 different filter materials at low (60–75 μg/L each) and high (325–350 μg/L) PFAS concentrations, respectively. Most strikingly, the nylon filters have the poorest overall recovery values for each of the six PFAS, while PP, GF, and MCE filters have mostly good recovery of all PFAS. At both PFAS concentration levels, all filter materials obey the following trend of adsorption: PFOS > PFNA > PFHxS ≅ PFOA > PFBS > GenX, suggesting that the competitive adsorption process is mostly similar regardless of the filter material (*i.e*., longer-chain PFAS adsorb more readily than their shorter-chain counterparts, PFSAs generally adsorb more readily than PFCAs).

**Figure 2:**
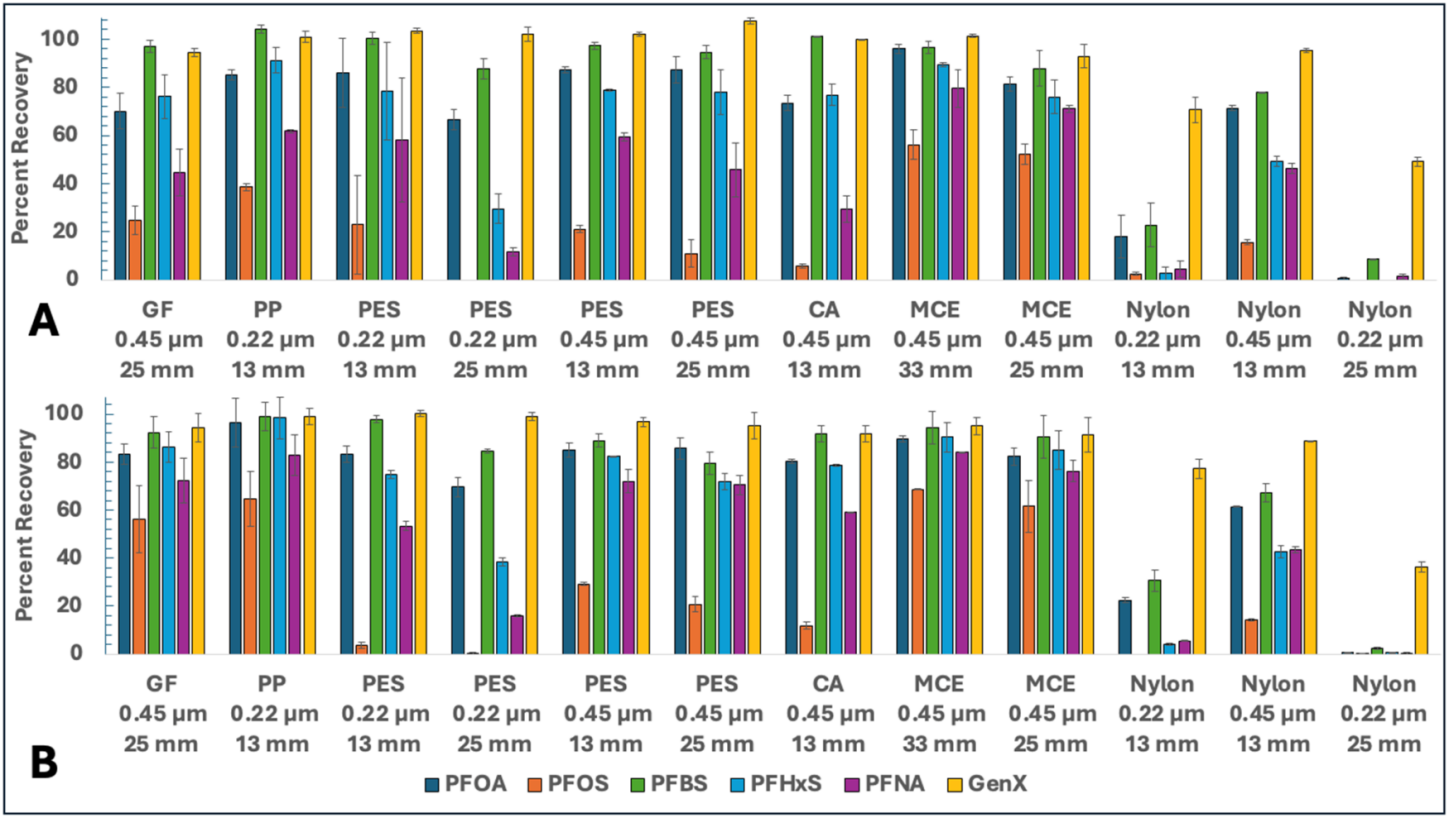
Percent recovery of each PFAS after filtration by each filter type. Conditions: DI water, (A) 60–75 μg/L of each PFAS, (B) 325–350 μg/L of each PFAS.

The effect of filter surface area, with higher adsorption occurring on greater available surface areas, is explored via variation in two material properties: (1) larger filter diameters (*e.g*., 13 mm vs 25 mm) and (2) pore size (*i.e*., 0.45 μm filters having lower overall surface area in comparison to comparable 0.22 μm filters). For both features, the filter with a larger surface area exhibits greater PFAS adsorption. This supports the use of filters with higher pore sizes and smaller diameters to minimize PFAS losses; however, the concentration and size of particles to be filtered should be considered to avoid filter clogging/passage of particles. These effects are specifically observed for the PES and nylon filters created by the same manufacturer (Samples B–H), adding substantial credence to the importance of filter selection when designing experiments.

The slightly higher percent recoveries of PFAS at a higher concentration can be attributed to adsorption dynamics and saturation effects: (1) finite numbers of adsorption sites where more PFAS can adsorb and (2) higher amounts of intermolecular interactions at higher concentrations to saturate adsorption sites. Even still, the differences in percent recovery at both concentrations are rather minor, which suggests that the overall adsorption capacity of the filters is not approaching its limit. Given the relatively short time of filtration (2 s), this might suggest kinetic limitations of the adsorption process, with equilibrium likely not being reached in such a quick time interval.

### 3.3 Effect of pH, Cations, Anions, and Matrix on Recovery

Based on performance differences attributed to surface area effects seen in Figure 2, the following filters, (A) PP 0.22 μm/13 mm, (D) PES 0.45 μm/13 mm, (F) CA 0.45 μm/13 mm, (H) Nylon 0.45 μm/13 mm, (K) MCE 0.45 μm/25 mm, and (L) GF 0.45 μm/25 mm, were used for further evaluation across a variety of different conditions entailing changes to pH, presence of cations or anions, and the tap water matrix effect. Importantly, these filters do not all share the same diameter, pore size, and overall surface area. Figure 3 shows the recovery of total PFAS (sum of six compounds) for each filter type across various test conditions. Recovery results for individual PFAS compounds associated with these tests are shown in SI Figures S4–S14.

**Figure 3:**
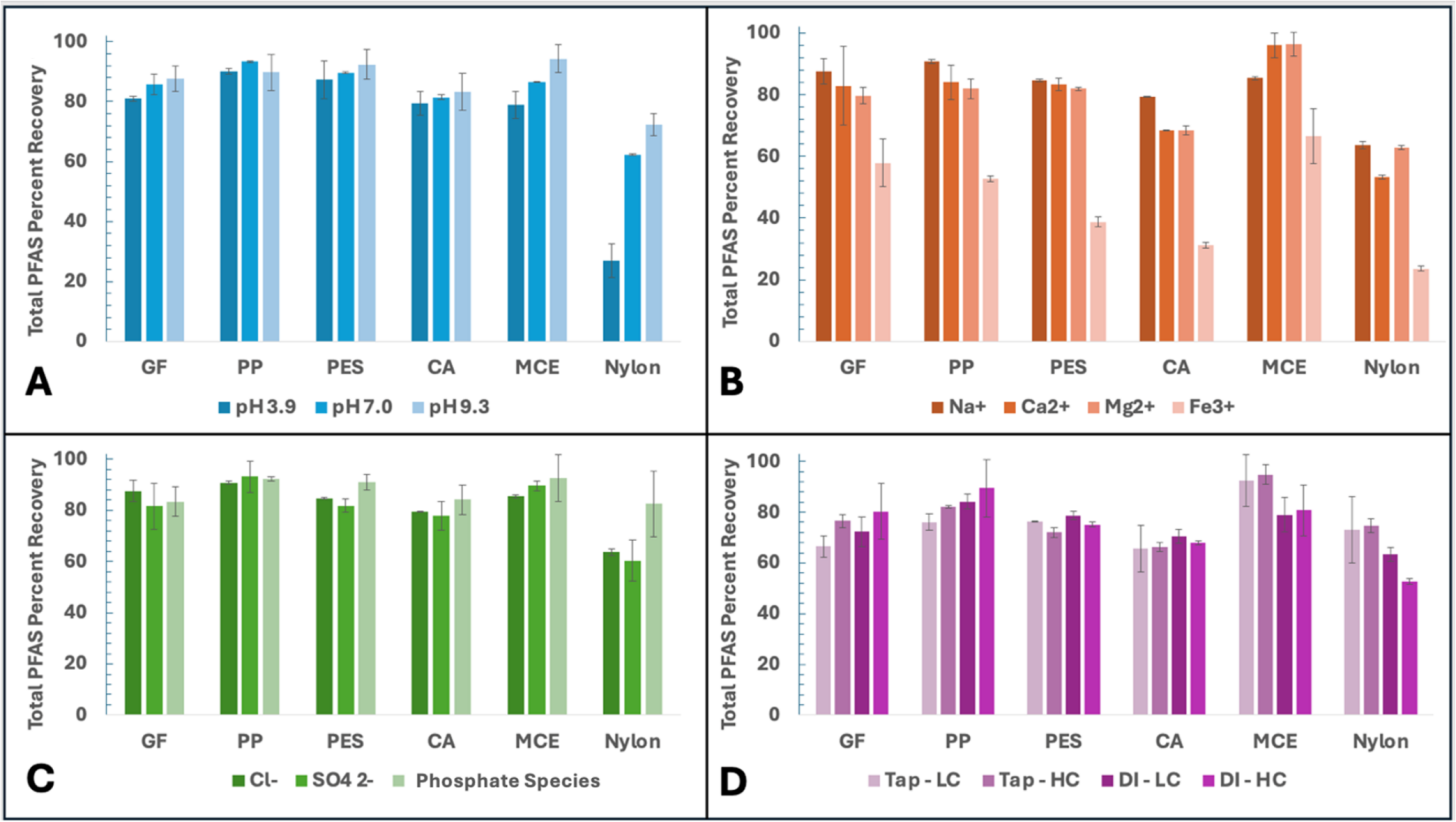
Recovery of total PFAS (sum of six compounds) by different filter materials. Effects of (A) pH, (B) cations (6 and 8 mg/L each), (C) anions (8 and 8 mg/L each), and (D) tap water versus DI water, high concentration (HC), and low concentration (LC). Conditions: 325–350 μg/L of each PFAS except for 60–75 μg/L of each PFAS in LC conditions, pH ∼ 6.1, except for pH-adjusted tests. Filters tested were A, D, F, H, K, and L according to Table 1.

Figure 3A shows the sum of total PFAS recovery for each membrane at pH 3.9, 7.0, and 9.3, with pH adjustment via either NaOH or HCl. For most filter types, total PFAS recovery is within experimental uncertainty across the different pH ranges to suggest this effect is insubstantial on overall recovery. It is evident that the nylon membranes have distinctly different performances as a result of solution pH, likely relating to their zeta potential and degree of overall amine protonation. The nylon filters contain both amide groups and amine groups (at the terminal ends of the polymer), the latter of which can be abundantly present due to polymer branching and different length segments. Amide groups do not deprotonate readily (>14 pH) and lack a positive charge; however, amines possess a p*K*_a_ of ∼9–10. Because of this, at lower pH, a greater percentage of the amine groups are positively charged (−16, −14, and −13 mV Zeta potential) compared to that at higher pH (−35, −36, −43 mV) (Table 2). Hence, nylon filter surfaces exert a greater electrostatic repulsion to anionic PFAS and increase recovery at higher pH.

The molar concentrations of cations used in the experiments were (in mM): Fe^3+^ = 0.143 (Cl^–^ counterion), Mg^2+^ = 0.329 (Cl^–^ counterion), Ca^2+^ = 0.150 (Cl^–^ counterion), and Na^+^ = 0.348 (Cl^–^ counterion). For the impacts of cations (Figure 3B), Fe^3+^ addition significantly reduces total PFAS recovery for all membrane types; this can be accredited to two effects—(1) the high positive charge density of Fe^3+^ decreases the overall negative surface charge of the membranes, subsequently reducing anionic PFAS repulsion, and (2) Fe^3+^ likely bridges PFAS anions to the negatively charged membrane surfaces, forming complexes that result in reduced passage through the membrane. While the divalent cations (Ca^2+^, Mg^2+^) have a less pronounced decrease in recovery compared to Fe^3+^, these effects are likely to have more influence in higher ionic strength conditions, which we discuss more in Section 3.4.2.

The molar concentrations of anions used in the experiments were (in mM) as follows: SO_4_^2–^ = 0.083 (Na^+^ counterion), total phosphate −H_2_PO_4_^–^, HPO_4_^2–^, and PO_4_^3–^ = 0.084 (Na^+^ counterion), and Cl^–^ = 0.226 (Na^+^ counterion). Interestingly, the presence of anions has little effect on PFAS recovery (Figure 3C), which is likely attributed to the inherently negative surface charges of the membranes. PO_4_^3–^ addition increases nylon’s overall recovery, which is probably caused by competition between PFAS and phosphate anions with the positively charged surface groups. Similarly to the cation case, at higher ionic strengths, it is expected that this anion effect on nylon’s recovery would be more pronounced.

Although there are some differences in molar concentrations and counterions among the tested salts and that phosphate exists as multiple charged species in the phosphate buffer system (not allowing for perfect isolation of ion identity effects), the data in Figure 3B,C still provide meaningful trends regarding the influence of ionic strength and specific ion types on PFAS recovery. The results should be viewed qualitatively rather than quantitatively,

Tap water’s influence is multifaceted (Figure 3D), as it is a more complex matrix compared to DI water, specifically, containing dissolved cations and anions, a slightly higher pH value of 7.1 compared to 6.1, and a higher concentration of dissolved organic carbon. Higher recoveries in tap water for the nylon and MCE filters are likely a function of solution pH, creating more negative overall surface charges at higher pHs, and interactions with cations. PP, which lacks ionizable organic functional groups, interestingly has decreased recovery in tap water compared to DI, which would suggest that cationic bridging effects occur between PFAS and its negatively charged surface.

### 3.4 Evaluation of Adsorption Mechanisms

#### 3.4.1 Abraham’s Solute Descriptors

Figure 4 depicts radial bar plots of the ASD (color-coded) predicted from RMG’s Solute ML method for six PFAS compounds and the dimers of each tested filter material (due to model limitations, monomers were used for MCE and PES); exact ASD values can be found in SI Table S2. Because few LFERs exist in the literature to predict the adsorption behavior of PFAS at water/polymeric solid interfaces and only recently have models begun to account for the unique properties of PFAS in models, the ASD data are interpreted qualitatively in this study, rather than applying them into LFERs for quantitative adsorption estimation. Herein, ASD values function as a comparative tool to provide molecular-level insights into the adsorption process. We also ignore the *L* and *V* parameters for the polymers because they are strongly associated with the number of monomers in an oligomer but less dependent on the polymer types.

**Figure 4:**
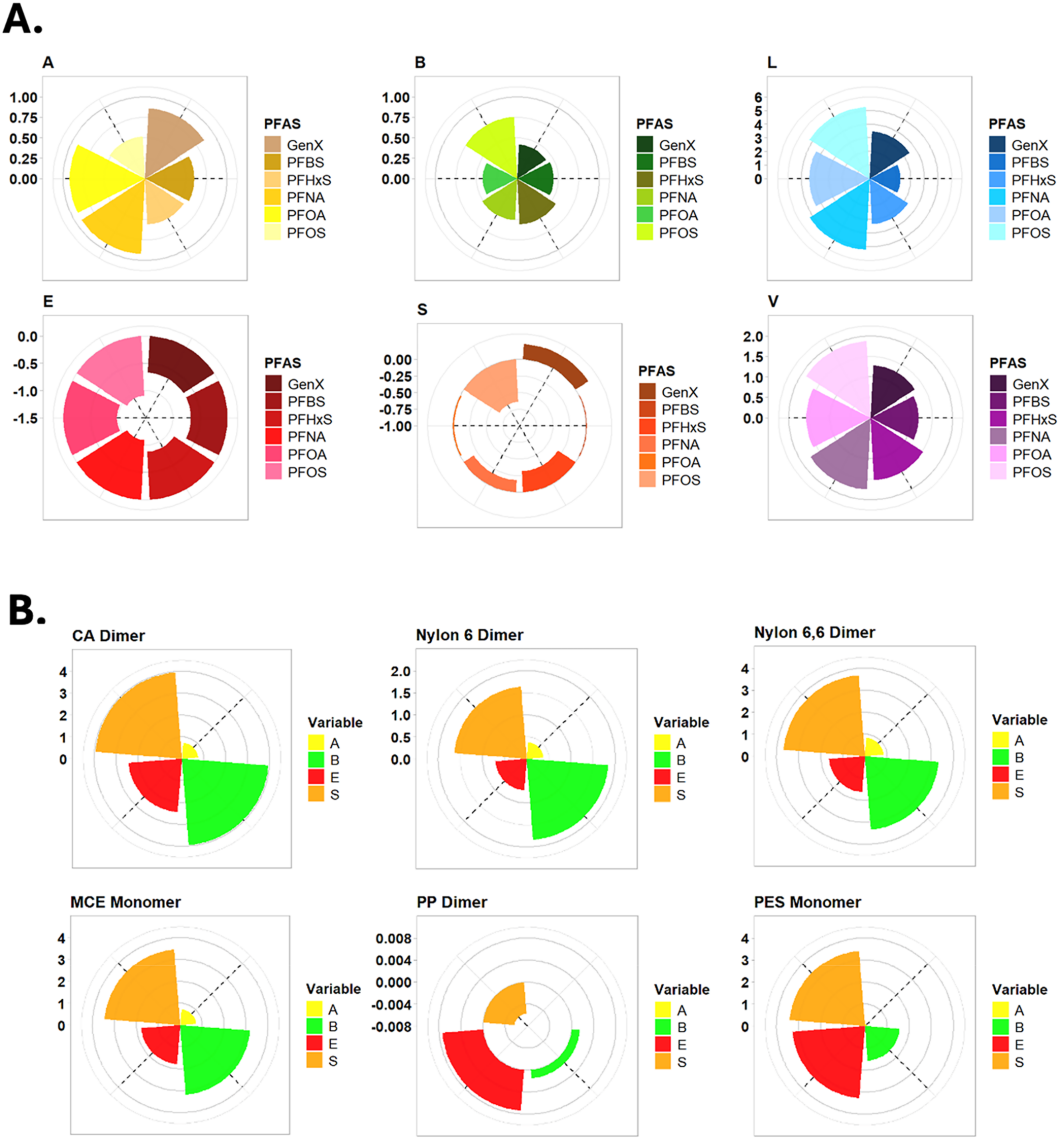
Predicted Abraham solute descriptors for (A) PFAS molecules and (B) filter materials. Conditions: dimers selected for the polymeric materials with the exception of PES and MCE. ASD is color-coded for translation across panels A to B.

Figure 4A compares individual PFAS categorized by each ASD. Expectedly, longer-chain PFAS molecules like PFNA and PFOS have greater log of gas-hexadecane partition coefficients (*L*, i.e., hydrophobicity) and McGowan characteristic volumes (*V*) compared to the shorter-chain ones, GenX and PFBS, which would encourage stronger hydrophobic interactions for long-chain PFAS. Regarding H-bonding ability, PFCAs (PFOA, PFNA, and GenX) are better H-donors (represented by *A*) than PFSAs (PFOS, PFHxS, PFBS), while PFSAs are better H-acceptors (represented by *B*) than PFCAs. As the chain length increases, the ability to accept H increases (*e.g*., the *B* values of PFOS and PFBS were predicted to be 0.755 and 0.444, respectively). It should be noted that since the RMG tool is unable to estimate ASD for ionic compounds, uncharged protonated PFAS structures were calculated in the model (instead of anionic deprotonated PFAS at environmentally relevant pH ranges). Despite the predicted differences in the *A* values, PFAS generally are poor H-bond donors due to their very low p*K*_a_ values and their fluorinated chains (SI Table S1). This suggests that the H-bond accepting potential of PFAS functional head groups would be of much greater importance for potential intermolecular interactions with available H-donors.

Fluorine’s inherent atomic nature bestows high electronegativity and low polarizability, which leads to generally low participation in van der Waals forces for the fluorinated chains. Fluorine forms significant dipoles when bonded to carbon atoms in comparison to carbon–hydrogen bonds (*i.e*., C–H: 0.4 D, C–F: 1.39 D); however, local dipole moments mostly cancel one another, rendering PFAS chains nonpolar and hydrophobic.^34,35^ Polarizability (represented by *S*) is related to both PFAS size and functional group; on average, PFAS of longer-chain length are less polarizable than that of their shorter-chain counterparts due to the presence of more fluorine atoms, and PFSAs are also less polarizable than PFCA counterparts (*e.g*., S = −0.020, −0.181, and −0.314 for PFOA, PFNA, and PFHxS, respectively), which would suggest stronger dipole–dipole interactions occurring for PFCAs. Excess molar refraction (represented by *E*), which reflects van der Waal forces and polarizability effects not covered by (S) (*e.g*., π–π interactions), is smaller for the longer-chain PFAS although generally similar for all compounds, which is reasonable, as the PFAS are fully saturated molecules that are only polarizable at their head groups.

Brown et al. described interesting trends regarding the prediction of *S* and *E* for PFAS, noting unconventionally negative and contrasting values for two sets of ASD for PFAS available in the literature, developed by Abraham and Endo.^36–38^ While ML algorithms have shown use in improving the predictive power of LSER models for PFAS adsorption,^39^ these discrepancies suggest that models need more experimental PFAS data incorporation to achieve more accurate predictions of these solute descriptors.

Figure 4B shows the predicted ASD values for dimers of the membrane materials, with the exception of PES and MCE, for which monomers were used due to model limitations. Due to differences in the size of polymers’ repeating unit sizes and because only monomers or dimers of the materials were used as inputs to the ML model, it is difficult to compare the predicted ASD values directly across materials, but these predictions still offer valuable insight into each material’s potential for intermolecular interaction. Repeating polymer units containing N or O atoms are good H-acceptors, and if they have an H atom attached to these atoms, they are also strong H-donors. In neutral pH conditions, anionic PFAS head groups act as H-acceptors and could participate in bonding with H-donors like CA, MCE, and nylon. CA and MCE, rich in hydroxyl and carboxyl groups, are the best H-donors of the filter materials. CA, MCE, nylon, and PES can all function as H-acceptors. Unsurprisingly, nylon-6,6, having an additional amide group in its repeating unit, is a better hydrogen donor and acceptor than that of nylon-6. N-containing groups (like nylon) are generally better H-acceptors than oxygenated groups due to their lower electronegativity and greater number of lone pair electrons.

MCE, CA, nylon, and PES are also all polarizable, as seen in *E* and *S*, so dipole–dipole interactions with polar PFAS carboxylic acid and sulfonic acid head groups are certainly possible. Comparing MCE monomer to CA monomer, MCE is slightly more polar due to the replacement of some acetyl groups with nitro groups, which would decrease its hydrophobicity and increase its water affinity. It is expected that PES would have the greatest molar refraction (*E*) of these materials having both aromatic, conjugated rings and sulfone groups; π electrons that contribute to (*E*) have been shown to be of importance for PFAS adsorption.^40^ In aqueous matrices, polarity and H-bonding ability present competing solvation with water molecules that could result in decreased PFAS adsorption. A study comparing the adsorption of PFAS onto pristine carbon nanotubes (CNTs) to ones functionalized with hydroxyl and carboxyl groups found less adsorption to occur onto the functionalized CNTs; the authors concluded that functionalized CNTs were more likely to form hydrogen bonds with water, decreasing sorption of PFAS, and that hydrophobic interactions were the principal driver of adsorption.^41^ The PP alkyl chain differs from the other test filter materials in that it is hydrophobic, lacks H-bond acidity and basicity, and is not polarizable, as reflected in its very low predicted ASD values. Subsequently, only Van der Waal forces and hydrophobic interactions are the primary viable mechanisms for PFAS adsorption on this material. RMG’s Solute ML method does not support silicon atoms in its calculations; therefore, hydrophilic GF filters, containing primarily silica, were not included here.

#### 3.4.2 Additional Contributors to Adsorption

The experimental data suggest that the PFAS adsorption phenomena are multifaceted and extend beyond the inherent properties of the PFAS and the membrane materials hypothesized from the ASD relationships. Despite the differences in the predicted ASD for both PFAS and polymer repeating units, the adsorption trend remained the same for all filter materials in all experimental conditions (PFOS > PFNA > PFOA ≅ PFHxS > PFBS > GenX), which agrees with other literature concerning the enhanced mobility and higher water affinity of shorter-chain PFAS compared to longer-chain ones. While the intermolecular forces described by the ASD likely have small roles contributing to the observed adsorption behavior, it is likely that the main drivers relate to (1) PFAS chain length and overall molecular size (*i.e*., hydrophobic interactions) and (2) electrostatic interactions.

Given the generally low recovery percentages of PFAS for the nylon filters across a variety of different experimental conditions, the effect of electrostatic interactions between cationic amine/amide groups and anionic PFAS is also highly important in engineering the adsorption of PFAS. Omorodion et al. conducted a DFT analysis and found that electrostatic interactions are the most prominent attractive forces in the context of PFAS intermolecular interactions although increasing chain length can allow for hydrophobic effects attributed to dispersion forces to become much more pronounced.^42^ Our previous work also shows a similar DFT calculation regarding the importance of electrostatic interactions for the adsorption of PFOA and PFBS onto powder-activated carbon functionalized with amino and pyrrolic moieties.^43^ These conclusions are concurrent with many other observations in the literature.^44,45^

From a physical perspective, there are clear linkages between surface area effects. Mercury intrusion, SEM, and AFM evidenced differences in total area, morphology, and topology among the filters. The higher roughness, strand-like filters ((A) PP and (L) GF) exhibited consistently good performance, which seems counterintuitive compared to smoother surfaces. It is unlikely that these differences play a significant role in short opportunities for adsorption to occur (such as single-use, short-contact time syringe filtration); however, in the context of a long-term membrane filtration process, roughness will most likely impact flux, fouling, and rejection efficiency.^46,47^

PFAS are known to form micelles or hemimicelles at high concentrations much above environmental levels (*e.g*., the critical micelle concentration (CMC) of PFOA in 100 mM NaCl is ∼3000 mg/L).^48^ Recently, it was found via joint experimental and molecular dynamics (MD) simulations that PFAS can self-assemble into micelles at concentration levels below the CMC at the surface of interfaces^49,50^ (*i.e*., localized high concentration micelle assembly). This unique property of PFAS could alter the available interactions that favor adsorption (*e.g*., shielding hydrophobic tails from interacting with membrane surfaces, encouraging greater PFAS–PFAS interactions) as well as create larger molecular structures that adsorb more readily or change the inherent adsorption behavior (e.g., blocking access to harder-to-reach active sites in the membrane surface). Shorter-chain PFAS have higher CMCs compared to longer-chain PFAS, which might contribute to the adsorption trends observed here.^51^ Additionally, the presence of ions might further encourage micelle formation, as the CMC of PFAS is lowered with increasing salt concentration due to decreased repulsive forces from anionic headgroups.^52^

The presence of multivalent ions, interestingly, has additional effects. Divalent and trivalent cations can “bridge” PFAS together via electrostatic interactions,^53^ subsequently making their molecular size larger, reducing their overall negative charge, and encouraging greater adsorption, especially onto negatively charged surfaces. Additionally, increasing ionic strength and valence in general compress the electrical double layer (EDL) of membrane surfaces, which can neutralize a surface’s zeta potential and could facilitate a higher degree of PFAS adsorption.^54^ Cai et al. studied the influence of Mg^2+^, Fe^3+^, and Ca^2+^ on PFAS sorption behavior of two different soils, finding that cationic presence neutralized the soils’ inherently negative zeta potential and facilitated higher PFAS sorption through decreased electrostatic repulsion, enhanced cationic bridging, and increased hydrophobicity.^55^

## 4 Conclusions and Environmental Implications

A myriad of research works in recent decades have measured PFAS concentrations in various matrices to elucidate their scientific understanding, particularly in environmental and public health contexts. In cases where syringe filtration must be utilized to protect sensitive analytical instrumentation from particle introduction or to filter contaminants from samples, care should be taken to understand the effects of PFAS adsorption on filter materials and optimize data quality. In this work, we demonstrate from an experimental and materials characterization perspective that not all filter materials are created equally (even when made by the same manufacturer) and that the adsorption phenomena are both complex and multifaceted.

Real-world environmental water samples undoubtedly bring chemical uncertainties and additional complexities to this filtration process. For instance, higher ionic strength matrices (*e.g*., hard groundwaters, brackish water) will greatly compress the EDL of membrane surfaces, thus weakening electrostatic repulsion and changing ion transport and surface interactions.^56^ Wastewater samples containing a complex composition of chemical oxygen demand (COD), particulates, and ions not typically found in drinking water (*e.g*., various metals, NH_4_^+^) could cause filter clogging, competition with adsorption sites, and different behavior patterns. GF filters are often used to prefilter wastewater samples, which could add an additional layer of PFAS removal when further filtration is utilized after pretreatment. Future studies could aim to further evaluate these effects across different matrices.

While we utilized RMG’s Solute ML method to predict ASD for various PFAS and polymer materials, we recognize that this methodology is only qualitatively beneficial to hypothesize adsorption mechanisms. As with any model, there exist inherent uncertainties in predictive power. Many LFER models are well-established, and ASD are available for thousands of different compounds; however, related information on PFAS has only recently been emerging in literature, mainly concerning fluorotelomer alcohols, fluoroethers, perfluoroalkanesulfonamides, *etc*., while availability is more limited regarding legacy PFCAs and PFSAs.^37^ Wider applicability and enhanced robustness of these models can be achieved through larger published data sets. Experimental measurements of additional adsorption data for PFAS at different interfaces will better train models to make more accurate predictions of adsorption behavior and a PFAS molecule’s true ASD.^57^ A current disadvantage of this approach is that existing LFER models are limited to neutral compounds and have not been trained for compounds that exist ionically at typical environmental pH ranges. Recently, Hatinoglu et al. used a modified LFER model incorporating a new ASD, *J’*, as an ionization parameter to estimate the adsorption behavior of various PFCAs onto polystyrene microplastics; this was in tune with suggested LFER modifications that account for ions, ionic species, and ion pairs, described by Abraham and Acree.^58,59^ Despite current limitations, ASD can lend useful qualitative information regarding molecular-level insights into chemical adsorption behavior.

Development of an “ideal” filtering material should strive to minimize potential intermolecular forces and matrix effects, while conversely, an “ideal” separation membrane should strive to maximize intermolecular interactions with targeted solutes like PFAS. While modeling efforts to examine the unique behavior of PFAS have been multilayered and have provided great knowledge to the field regarding PFAS adsorption onto different surfaces, there exists a further need to explore these relationships from additional angles, such as incorporating PFAS-containing nonconventional functional groups and the copresence of PFAS mixtures with other water contaminants. ML and computational simulations will certainly be immensely useful to explore this realm, especially from an inverse design perspective.^60,61^ For instance, ML models combined with Bayesian optimization were able to identify membrane monomers and fabrication conditions that could theoretically break the upper bound for membrane water/salt selectivity and permeability; experimental synthesis and testing of these novel membranes provided validation.^62^

We conclude by elevating PP, MCE, and GF filters as likely the best commercially available candidates to utilize when working with most PFCAs and PFSAs although losses to a certain degree are inevitable. Syringe filter membranes with larger pore sizes and smaller diameters are preferable. Since other studies have found limitations for these materials under other experimental conditions, incorporating a screening step to evaluate multiple filter candidates during experimental design is recommended, and the specificity of performance based on unique experimental conditions should be acknowledged. Beyond enhancing analytical procedures, this work also offers insights relevant to membrane fabrication and design.

## Supplementary Material



## Notes

PFAS molecular structure and physicochemical properties (Table S1); chemical structures of the filter materials (Figure S1); MIP data for PES 0.22 μm, 13 mm and PES 0.45 μm, 13 mm (Table S2); SEM images of filters not shown in main text (Figure S2); AFM images of the filters not shown in main text (Figure S3); percent recovery of all PFAS for best performing filters—pH 7 (Figure S4); percent recovery of all PFAS for best performing filters—pH 9.3 (Figure S5); percent recovery of all PFAS for best performing filters—pH 3.9 (Figure S6); percent recovery of all PFAS for best performing filters—8 mg/L NaCl (Figure S7); percent recovery of all PFAS for best performing filters—6 mg/L Ca^2+^ (Figure S8); percent recovery of all PFAS for best performing filters—8 mg/L Mg^2+^ (Figure S9); percent recovery of all PFAS for best performing filters—8 mg/L Fe^3+^ (Figure S10); percent recovery of all PFAS for best performing filters—8 mg/L PO_4_^3–^ (Figure S11); percent recovery of all PFAS for best performing filters—8 mg/L SO_4_^2–^ (Figure S12); percent recovery of all PFAS for best performing filters—tap water, low PFAS concentration (Figure S13); percent recovery of all PFAS for best performing filters—tap water, high PFAS concentration (Figure S14); Abraham’s solute descriptor values for PFAS and monomers/dimers of the test polymers predicted by RMG Solvation Tools Solute ML Method (Table S3) (PDF)
