# Supplementary material for: Insights on the Adsorption of Per- and Polyfluoroalkyl Substances onto Laboratory Syringe Membrane Filters: Experimental, Materials, and Mechanism Evaluations
Source: ACS ES T Water. 2026 Mar 7;6(4):2047–58. doi: 10.1021/acsestwater.5c00438 (PMC13077688; doi:10.1021/acsestwater.5c00438)
Supplement: Supplementary file 1 [file ew5c00438_si_001.pdf]

SUPPORTING INFORMATION

Insights on the Adsorption of Per- and Polyfluoroalkyl Substances onto Laboratory Syringe Membrane Filters: Experimental, Materials, and Mechanism Evaluations

Elliot Reid,[1] Qingquan Ma,[1] Thomas Igou,[2] Ching-Hua Huang,[1] and Yongsheng Chen[1,*]

[1] School of Civil and Environmental Engineering, Georgia Institute of Technology, Atlanta, Georgia, 30332, United States

[2] Water Tectonics, 6300 Merrill Creek Parkway Ste C100, Everett, Washington, 98203, United States

[*]**Corresponding Authors**: ching-hua.huang@ce.gatech.edu; yongsheng.chen@ce.gatech.edu

Number of Figures – 12

Number of Tables – 3

Number of References – 3

Number of Pages - 10

Table S1 – PFAS Molecular Structure and Physicochemical Properties

PFAS Name	Structure	$LogD_{OW}$ (pH = 7)	m/z	Molecular Volume (cm^3/mol)	Charge Density (meq/g C)	pK_a
PFOA	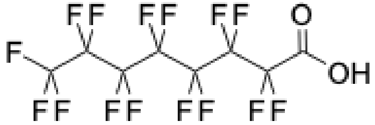	4.34	413 -> 169	226	-10.42	-0.21
PFOS	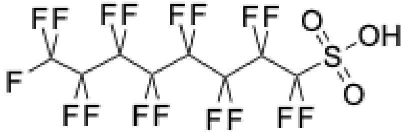	3.47	499 -> 79.9	256	-10.42	0.14
PFBS	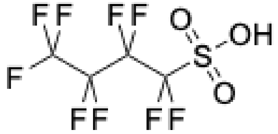	-0.97	299 -> 79.9	151	-20.83	0.14
PFNA	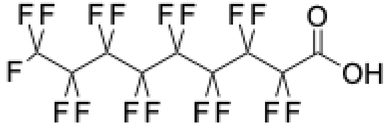	5.59	463 -> 419	253	-9.26	-0.21
PFHxS	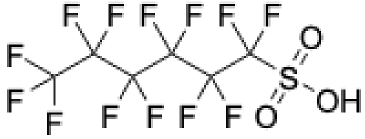	1.12	399 -> 79.9	202	-13.89	0.14
GenX	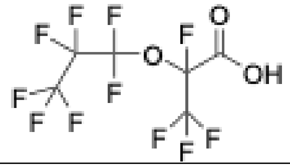	1.34	285 -> 185	184	-13.9	-0.06

Data from – Gagliano *et al.*, 2020, Liu and Sun, 2021, Kancharla *et al.*, 2022

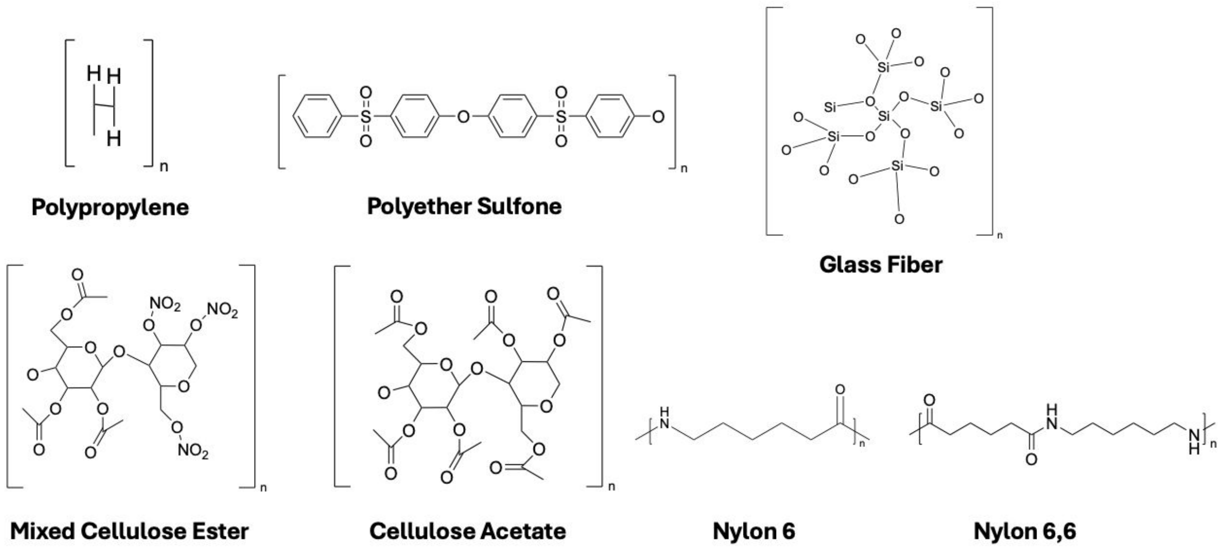

Figure S1 – Chemical structures of the filter materials

Table S2 – Mercury intrusion data for (B) PES 0.22 µm, 13 mm and (C) PES 0.45µm, 13 mm

Parameter	(B) PES 0.22 µm, 13 mm	(D) PES 0.45µm, 13 mm
Total intrusion volume at 59,959 psia (mL/g)	2.6839	2.3761
Total pore area at 59,959 psia (m^2/g)	61.824	49.088
Median pore diameter (volume) at 143.76 psia and 0.514 mL/g (µm)	0.7848	1.1538
Median pore diameter (area) at 20,516.42 psia and 11.060 m^2/g (µm)	0.00797	0.00642
Average pore diameter (4V/A) (µm)	0.17365	0.1936

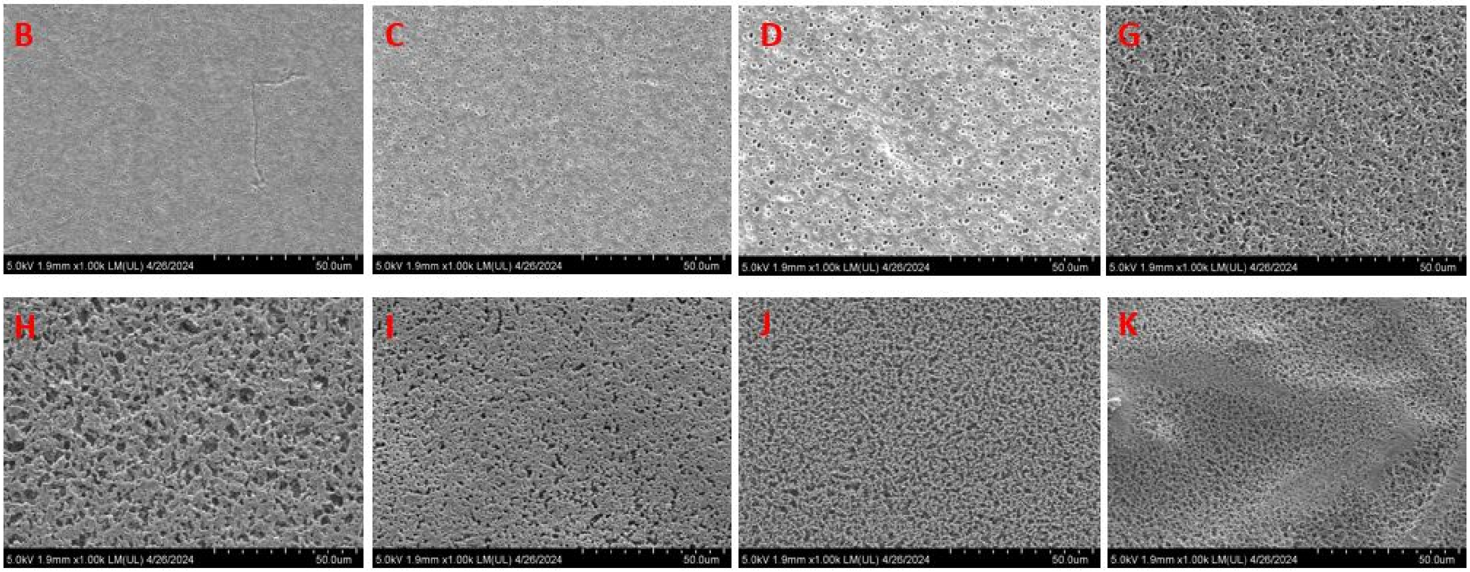

Figure S2 – SEM Images of the filters not shown in the main text (50 µm resolution). (B) PES, 13 mm, 0.22 µm, (C) PES, 13 mm, 0.45 µm, (D) PES, 25 mm, 0.22 µm, (G) Nylon, 13 mm, 0.22 µm, (H) Nylon, 13 mm, 0.45 µm, (I) Nylon, 25 mm, 0.22 µm, (J) MCE, 33 mm, 0.45 µm, and (K) MCE, 25 mm, 0.45 µm

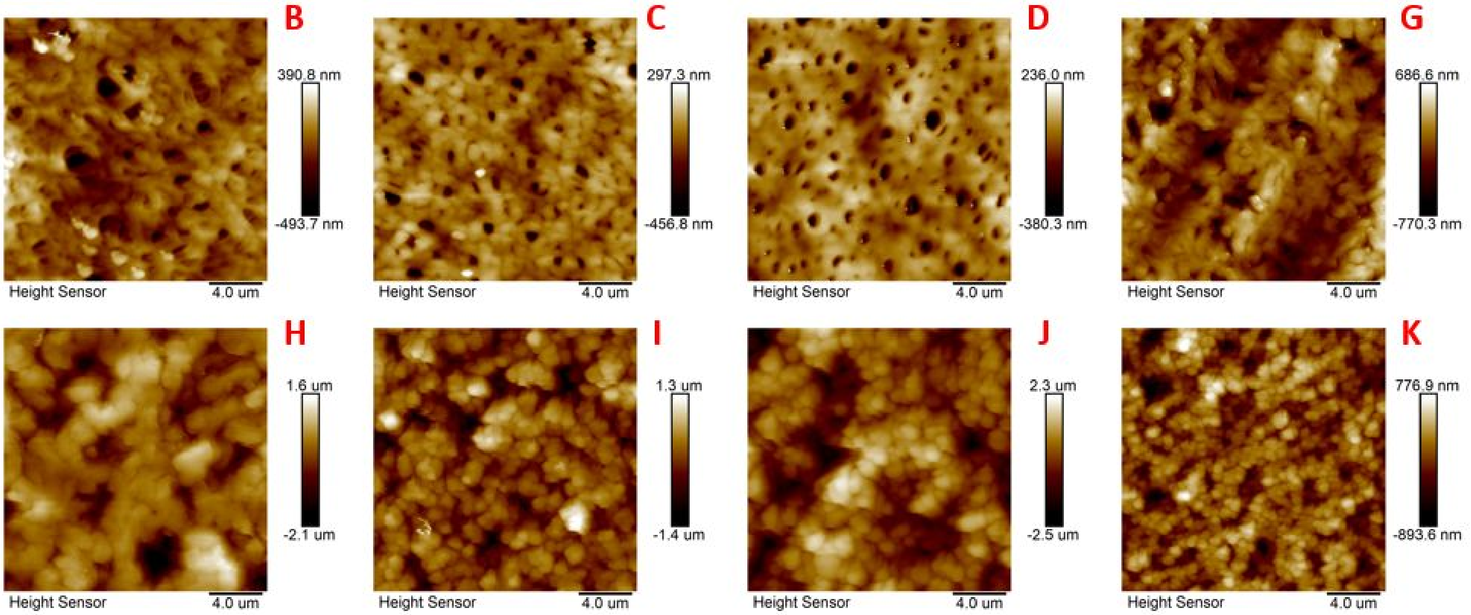

Figure S3 – AFM Images of the filters not shown in the main text. (B) PES, 13 mm, 0.22 µm, (C) PES, 13 mm, 0.45 µm, (D) PES, 25 mm, 0.22 µm, (G) Nylon, 13 mm, 0.22 µm, (H) Nylon, 13 mm, 0.45 µm, (I) Nylon, 25 mm, 0.22 µm, (J) MCE, 33 mm, 0.45 µm, and (K) MCE, 25 mm, 0.45 µm

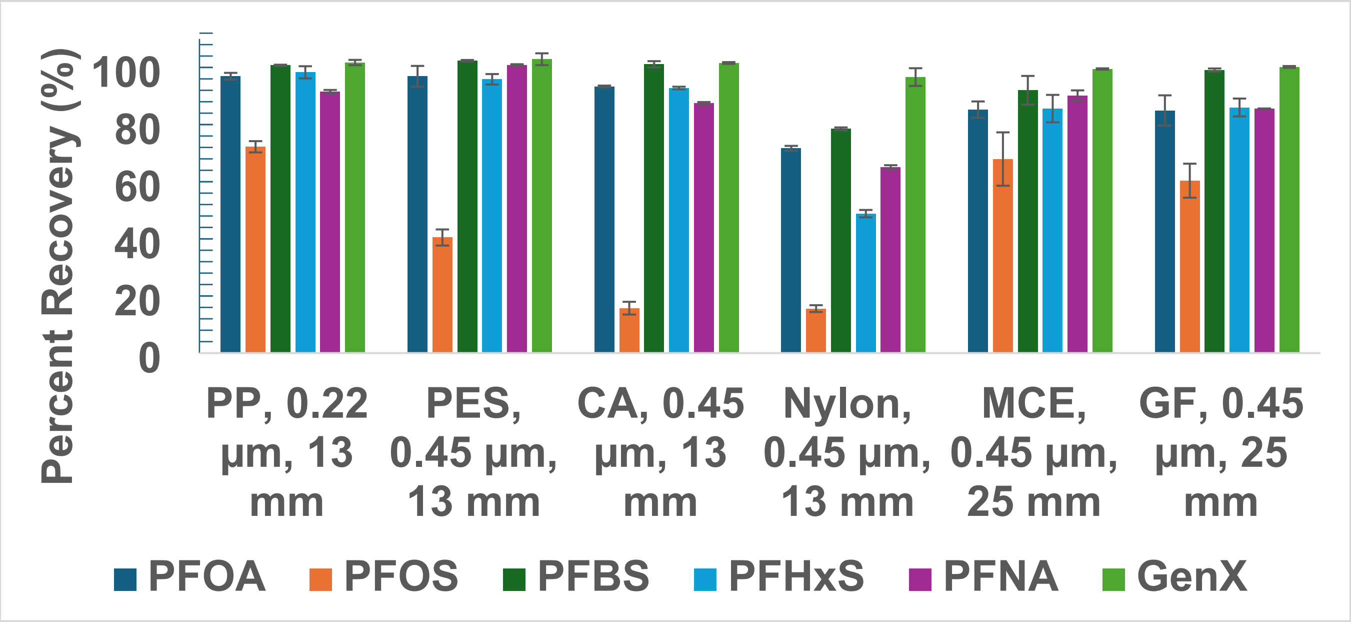

Figure S4 – Percent Recovery of all PFAS for best-performing filters – Conditions: Deionized Water, Adjusted to pH 7.0 with HCl, 300 ppb of each PFAS

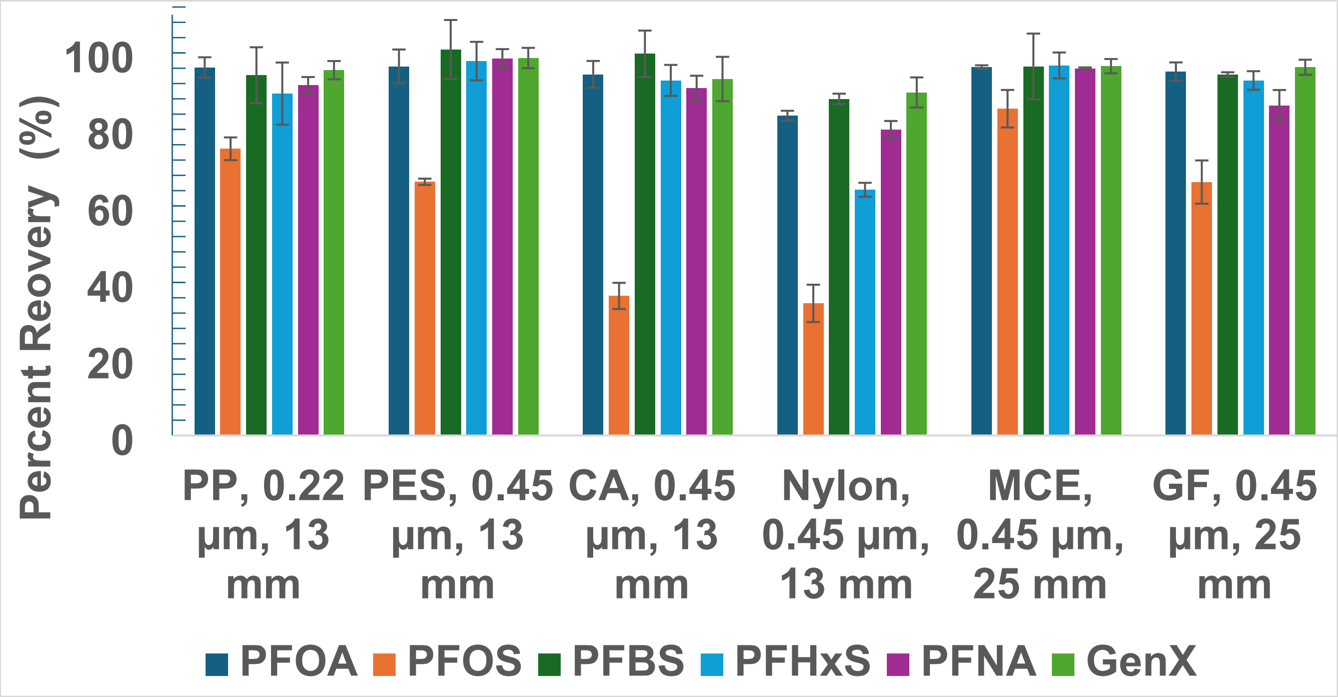

Figure S5 – Percent Recovery of all PFAS for best-performing filters – Conditions: Deionized Water, Adjusted to pH 9.3 with NaOH, 300 ppb of each PFAS

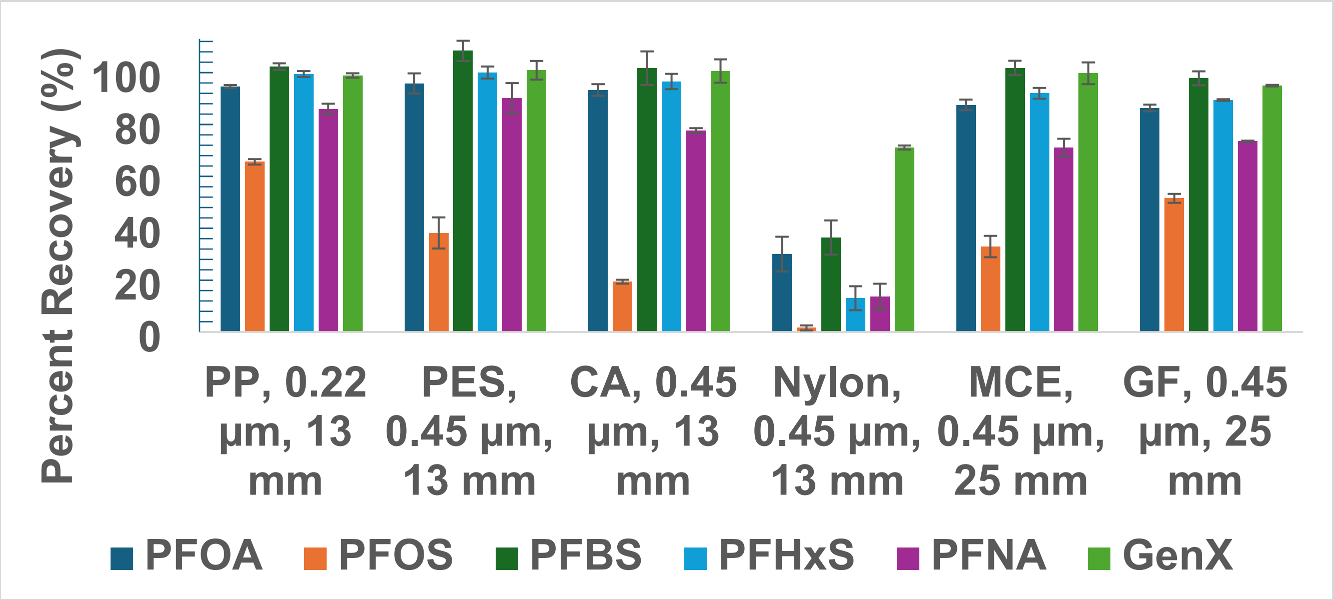

Figure S6 – Percent Recovery of all PFAS for best-performing filters – Conditions: Deionized Water, Adjusted to pH 3.9 with HCl, 300 ppb of each PFAS

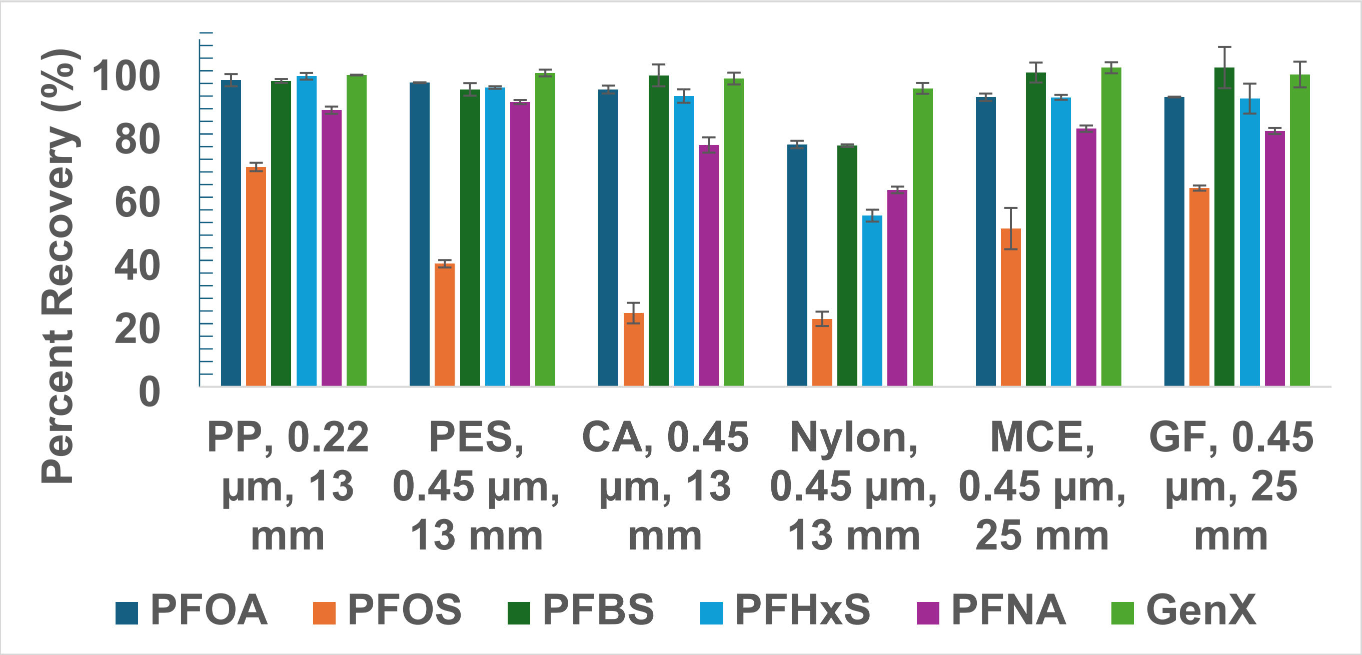

Figure S7 – Percent Recovery of all PFAS for best-performing filters – Conditions: Deionized Water, Addition of 8 mg/L NaCl, 300 ppb of each PFAS

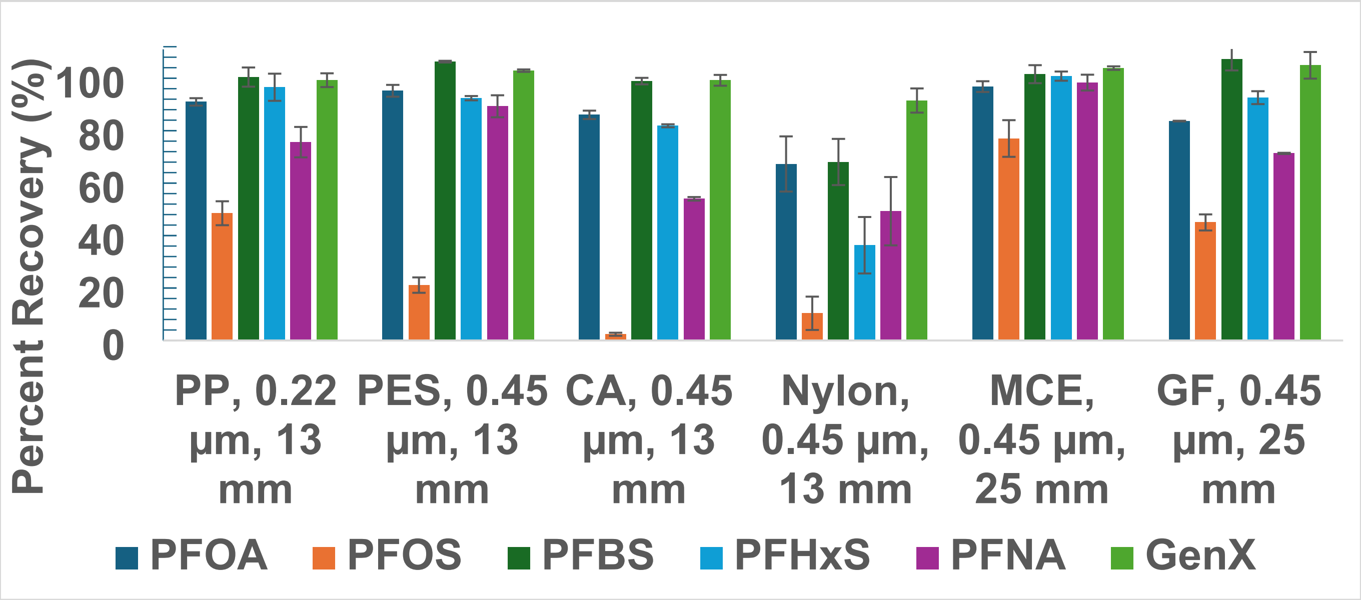

Figure S8 – Percent Recovery of all PFAS for best-performing filters – Conditions: Deionized Water, Addition of 6 mg/L Ca^{2+}, 300 ppb of each PFAS

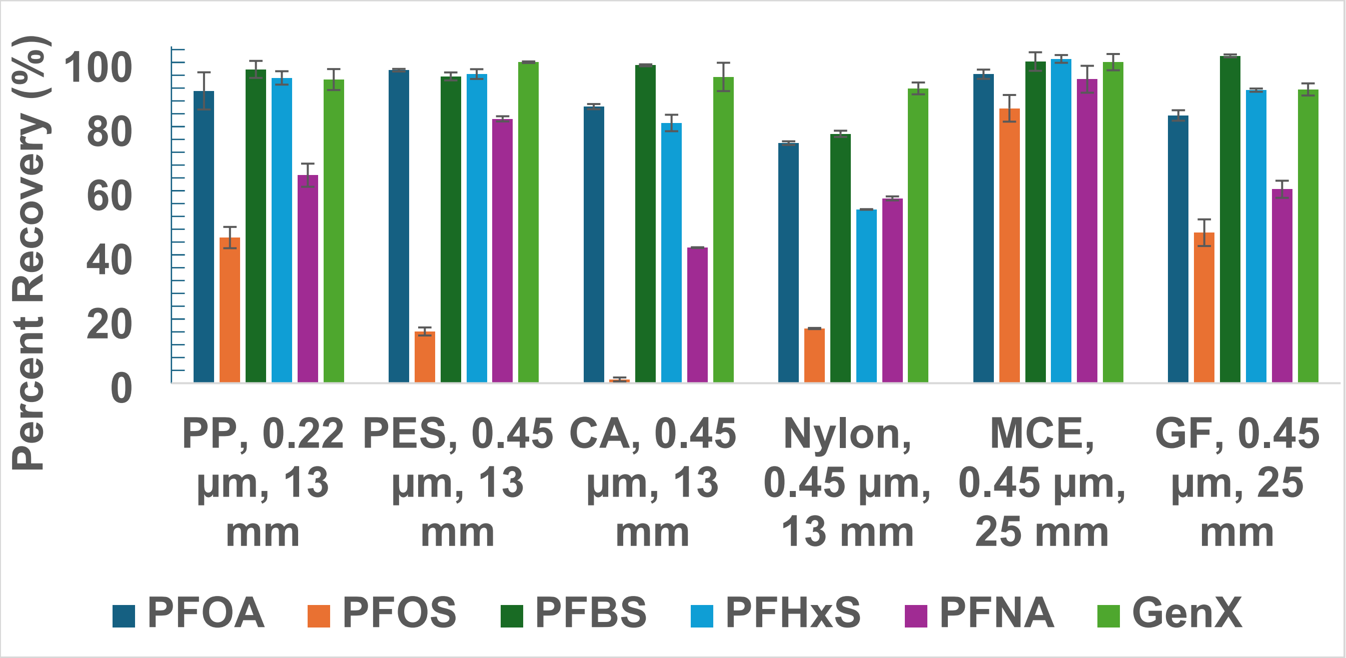

Figure S9 – Percent Recovery of all PFAS for best-performing filters – Conditions: Deionized Water, Addition of 8 mg/L Mg^{2+}, 300 ppb of each PFAS

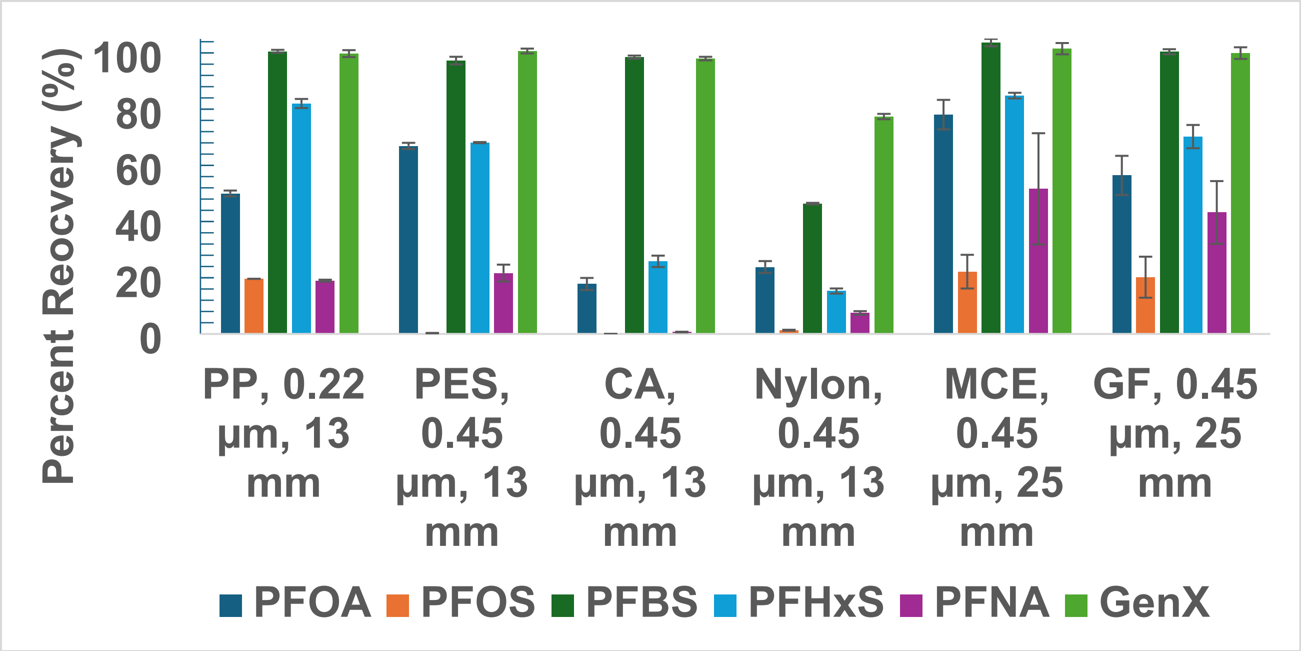

Figure S10 – Percent Recovery of all PFAS for best-performing filters – Conditions: Deionized Water, Addition of 8 mg/L Fe^{3+}, 300 ppb of each PFAS

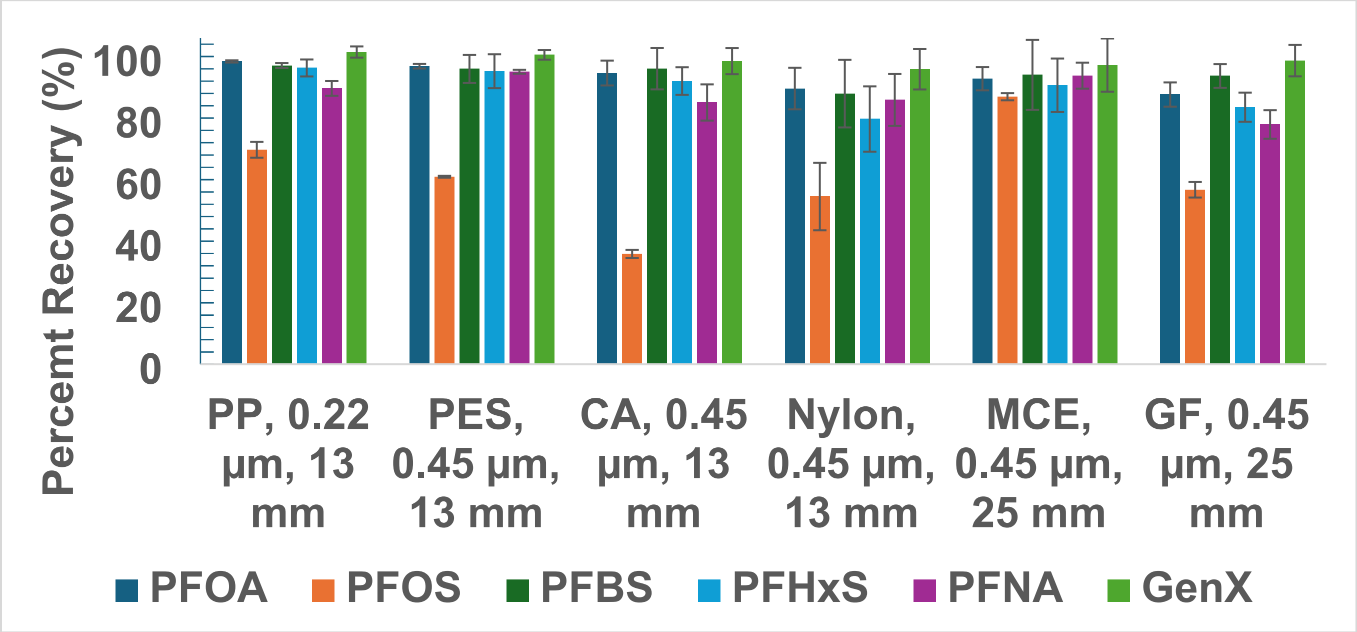

Figure S11 – Percent Recovery of all PFAS for best-performing filters – Conditions: Deionized Water, Addition of 8 mg/L PO_4^{3-}, 300 ppb of each PFAS

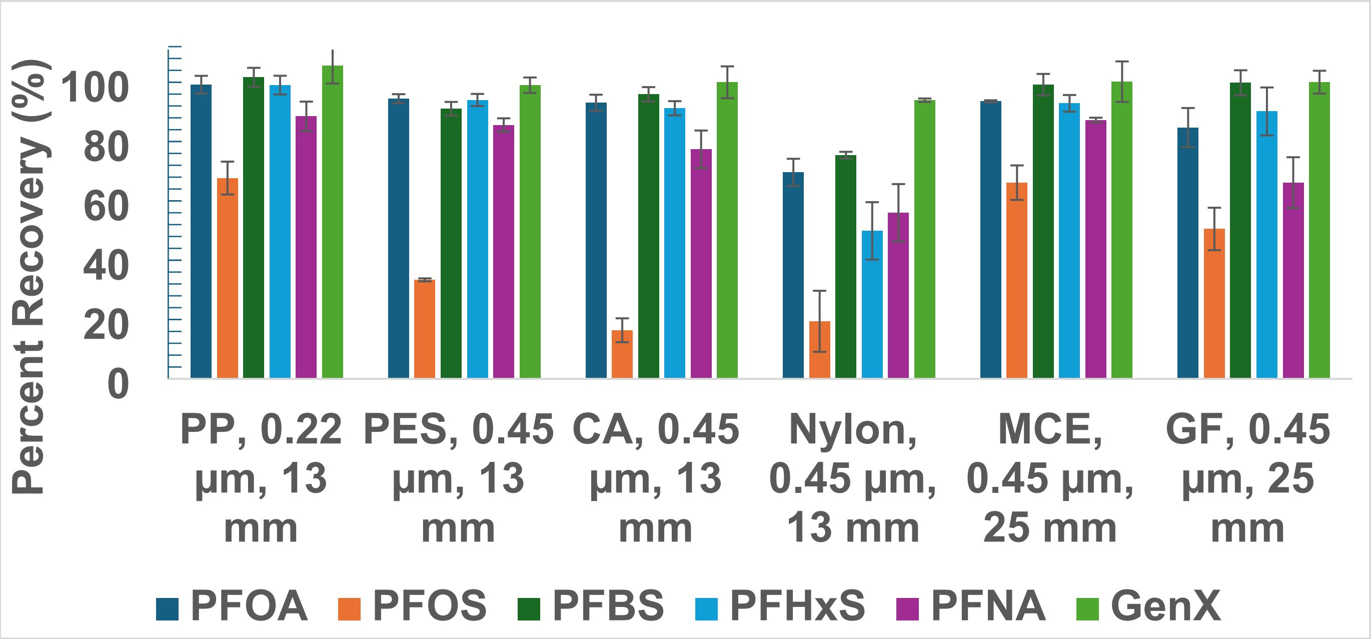

Figure S12 – Percent Recovery of all PFAS for best-performing filters – Conditions: Deionized Water, Addition of 8 mg/L SO_4^{2-}, 300 ppb of each PFAS

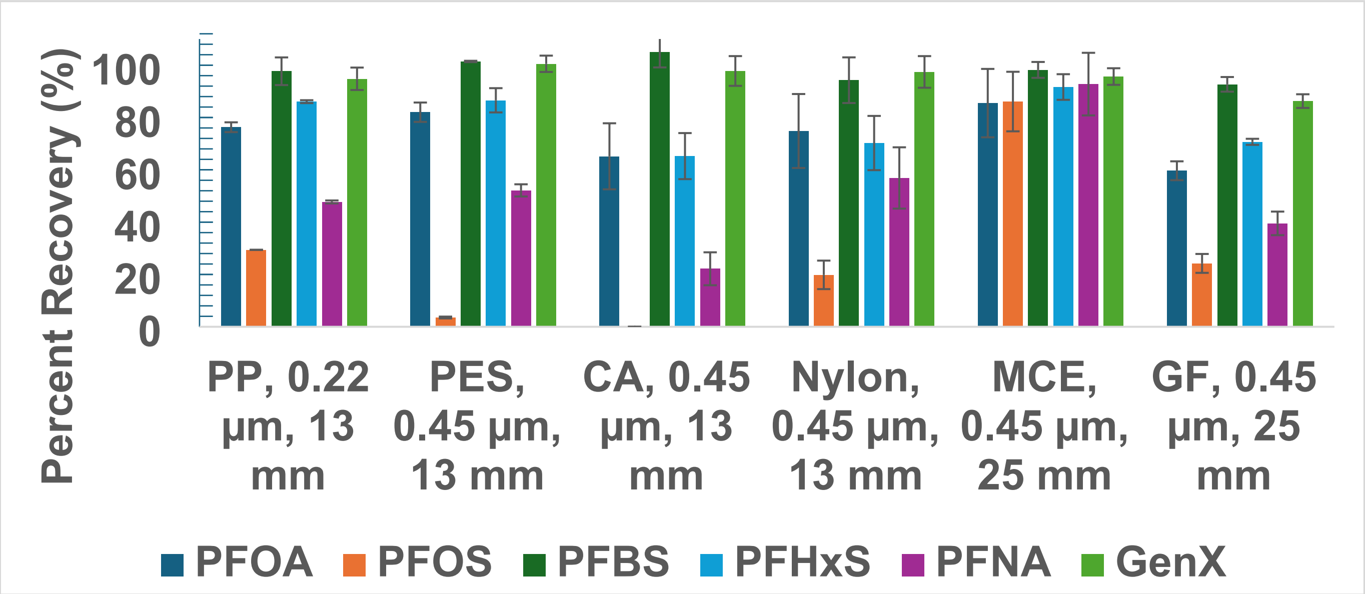

Figure S13 – Percent Recovery of all PFAS for best-performing filters – Conditions: Tap Water, Addition of 75 ppb each PFAS

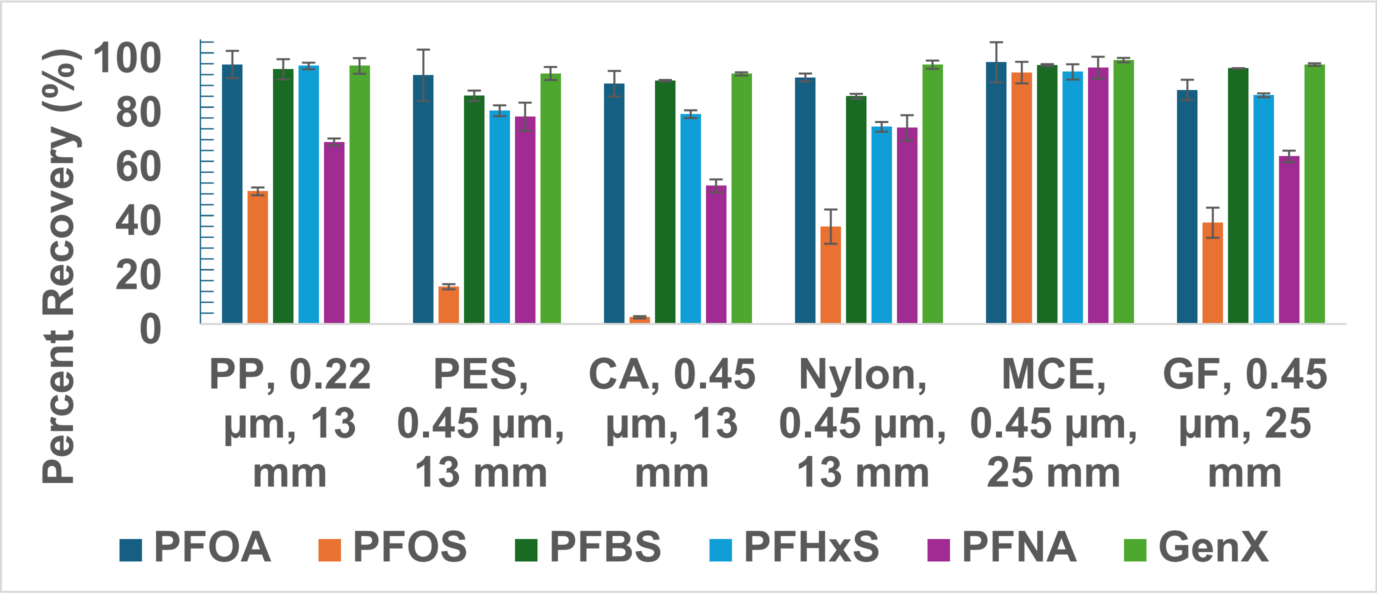

Figure S14 – Percent Recovery of all PFAS for best-performing filters – Conditions: Tap Water, Addition of 300 ppb each PFAS

Table S3 - Abraham's solute descriptors for PFAS and monomers/dimers of test polymer candidates generated by RMG Solvation Tools Solute ML Method

Test Candidate		E	S	A	B	L	V
PFOA		-0.975	-0.020	0.921	0.422	4.423	1.574
PFOS		-1.092	-0.638	0.512	0.755	5.255	1.875
PFHxS		-0.881	-0.314	0.561	0.560	3.332	1.522
PFBS		-0.669	0.010	0.601	0.444	2.263	1.170
PFNA		-1.093	-0.181	0.923	0.508	5.223	1.750
GenX		-0.672	0.230	0.860	0.418	3.466	1.281
Nylon 6	Monomer	0.217	1.260	0.284	0.538	4.444	1.0480
	Dimer	0.712	1.643	0.380	1.844	10.16	2.453
Nylon 6,6	Monomer	0.731	2.111	0.429	1.686	9.760	2.171
	Dimer	1.633	3.685	0.840	3.336	19.94	4.093
Polypropylene – Monomer		0.011	-0.13	-0.002	0.003	1.031	0.531

Dimer	0.007	-0.006	0	0.002	2.535	0.954
Cellulose Acetate - Monomer	1.502	3.347	0.527	3.3262	19.87	3.93
Dimer	2.449	3.956	0.739	3.950	24.86	7.50
Mixed Cellulose Ester - Monomer	1.778	3.462	0.729	3.187	19.534	3.563
Polyether sulfone - Monomer	3.284	3.414	0.109	1.5733	19.634	3.1975

References

Gagliano, E. *et al*. Removal of poly- and perfluoroalkyl substances (PFAS) from water by adsorption: Role of PFAS chain length, effect of organic matter, and challenges in adsorbent regeneration. *Water Research*, **2020**, 171, 115381

Kancharla, S. Alexandridis, P. Tsianou, M. Sequestration of poly- and perfluoroalkyl substances (PFAS) by adsorption: surfactant and surface aspects. *Current Opinion in Colloid and Interface Science*, **2022**, 58, 101571

Liu, Y.L. Sun, M. Ion exchange removal and resin regeneration to treat per- and polyfluoroalkyl ether acids and other emerging PFAS in drinking water. *Water Research*, **2021**, 207, 117781
